# The Heat Treatment Influence on the Microstructure and Hardness of TC4 Titanium Alloy Manufactured via Selective Laser Melting

**DOI:** 10.3390/ma11081318

**Published:** 2018-07-30

**Authors:** Zhan-Yong Zhao, Liang Li, Pei-Kang Bai, Yang Jin, Li-Yun Wu, Jing Li, Ren-Guo Guan, Hong-Qiao Qu

**Affiliations:** 1 School of Materials Science and Engineering, North University of China, Taiyuan 030051, China; syuzzy@126.com (Z.-Y.Z.); nucliliang@126.com (L.L.); nucjinyang@126.com (Y.J.); wuliyunnuc@126.com (L.-Y.W.); jing.li3d@hotmail.com (J.L.); nucqhq1993@126.com (H.-Q.Q.); 2 School of Materials Science and Engineering, Northwestern Polytechnical University, Xi’an 710072, China; guanrg@smm.neu.edu.cn

**Keywords:** selective laser melting, titanium alloy, heat treatment, microstructure, microhardness measurement

## Abstract

In this research, the effect of several heat treatments on the microstructure and microhardness of TC4 (Ti6Al4V) titanium alloy processed by selective laser melting (SLM) is studied. The results showed that the original acicular martensite α′-phase in the TC4 alloy formed by SLM is converted into a lamellar mixture of α + β for heat treatment temperatures below the critical temperature (*T*_0_ at approximately 893 °C). With the increase of heat treatment temperature, the size of the lamellar mixture structure inside of the TC4 part gradually grows. When the heat treatment temperature is above *T*_0_, because the cooling rate is relatively steep, the β-phase recrystallization transforms into a compact secondary α-phase, and a basketweave structure can be found because the primary α-phase develop and connect or cross each other with different orientations. The residence time for TC4 SLM parts when the treatment temperature is below the critical temperature has little influence: both the α-phase and the β-phase will tend to coarsen but hinder each other, thereby limiting grain growth. The microhardness gradually decreases with increasing temperature when the TC4 SLM part is treated below the critical temperature. Conversely, the microhardness increases significantly with increasing temperature when the TC4 SLM part is treated above the critical temperature.

## 1. Introduction

Selective laser melting (SLM) is a layer-based deposition method using a laser to selectively melt successive layers of metal powder in an inert-gas-filled chamber [1,2]. SLM can quickly and accurately produce metallic components of any complex shape on the SLM equipment through 3D CAD data directly [3,4,5]. In the SLM, the 3D CAD model is imported into the SLM software system and afterwards sliced into layers with a certain thickness [3]. In the manufacturing process, the parameters such as the power of laser previously defined is applied and successive layers of powder is melts and forms a liquid pool by the interaction of a laser beam [6]. The molten pool will solidifies and cools down quickly [6,7,8]. Then, successive layers of powders are deposited, each one corresponding to a slice of the 3D CAD model [9]. Compared with traditional production technology, it provides a better alternative in certain aspects, such as a reduction of production steps, a high level of flexibility, high material use efficiency, and a near-net-shaped production for the manufacture of complex geometries [10,11,12]. SLM has been widely used in the fields of medical, military, aerospace, and automobile manufacturing [13].

Titanium alloys such as TC4 have been widely used in both the biomedical and aerospace industries, due to their high weight-to-strength ratio and superior mechanical properties, i.e., high strength, high corrosion resistance, and fracture toughness [14,15,16]. Extensive previous research has focused on titanium produced by SLM. For instance, Thijs et al. studied the influence of scanning parameters and scanning strategy on the microstructure of TC4 alloy. They found that the microstructure of TC4 alloy processed by SLM is the acicular martensitic phase due to the higher temperature gradient during the SLM process; furthermore, due to the occurrence of epitaxial growth, elongated grains emerge [17]. The direction of grain growth is mainly related to the local heat conduction determined by the scanning strategy; more specifically, the grain direction is parallel with the local conductive heat transfer [17]. Bin Zhou et al. prepared TC4 alloy through SLM under vacuum conditions. Their test results showed that this process could reduce the porosity of Ti6Al4V samples [18]. Investigating the effect of build orientation of TC4 manufactured by SLM, Agius et al. revealed a unique α′ martensite microstructure and identified an asymmetric cyclic softening phenomenon [19]. Moreover, the residual stresses presented in the SLM coupons had a significant influence on the cyclic behavior of the material.

TC4 is a typical double-phase titanium alloy. The sharp cycles of steep heating and cooling rates in the SLM process resulted in typical TC4 part internal microstructures that consist of prior β columnar grains filled with acicular a’ martensite [3,6,8,17]. Thus, the TC4 titanium alloy prepared by SLM usually displays high yield strength, but limited ductility and low fatigue resistance, therefore making it unsuitable for structural applications [20,21]. Heat treatment has an important effect on the properties of SLM-formed TC4 alloy. Vrancken et al. studied the effect of this process on the alloy’s mechanical properties and microstructure. Their results shows that the original martensite α′-phase is converted to a lamellar mixture of α and β following heat treatment temperatures, but features of the original microstructure are maintained [22]. Yao et al. studied the influence of heat treatment on the microstructure evolution of as-built 3D laser-deposited TC4 alloy, and examined the mechanical behaviors in detail; they proved that the heat treatment could improve both the yield stress and the compressive strength of the 3D laser-deposited TC4 alloy [23]. Dai et al. reported the corresponding relationship between the heat treatment temperature and the corrosion resistance of SLM-formed TC4 titanium alloy, and found a negative effect [24], while Young-Kyun Kim et al. improved the creep resistance through heat treatment only [25]. Gerrit et al. studied the effects of extensive heat treatment temperatures on the phase transformation (phase fraction) and tensile properties of SLM-formed TC4 titanium alloys [26]. Furthermore, Wu et al. established the corresponding regular patterns of microstructure and hardness with heat treatment temperature [27]. Their results indicate that the heat treatment can effectively improve the tensile properties and hardness of SLM formed TC4 titanium alloy [26,27]. Therefore, heat treatment can effectively control the microstructure of TC4 alloy after SLM forming. It can eliminate the residual stress and improve its mechanical properties, which is important for improving the fatigue strength and service life of the alloy.

Owing to the β→α′ transformation at rapid cooling rates, TC4 under SLM processing typically results in extensive formation of brittle martensitic microstructures. The appropriate heat treatment process can release the residual stress in the SLM process; control the α − β phase transformation; adjust the shape, size and content of the phase; and optimize the microstructure and mechanical properties. Two temperature regions divided by the critical temperature (*T*_0_) are used in this study: below the *T*_0_ region (BTR) and above the *T*_0_ region (ATR). The critical temperature is defined as the temperature above which the titanium alloy forms the α′-phase during rapid cooling (water quenching or air cooling). This temperature *T*_0_ is calculated based on a thermodynamic database by Lu et al. [28] and Ji et al. [29], who identified *T*_0_ as 893 °C and 872 °C, respectively. Therefore, based on previous research, the SLM-formed TC4 titanium alloy was post-treated by a wide range of heat treatment temperatures; the typical microstructure was obtained and the change of hardness was revealed. This study can thus provide recommendations for the preparation of high performance SLM TC4 alloy.

## 2. Materials and Methods

**Figure 1:**
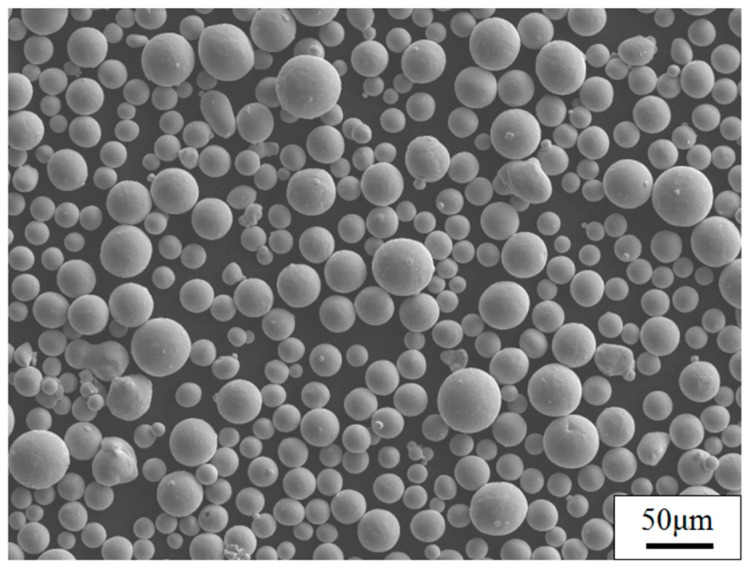
SEM image of TC4 powders.

Cuboidal shape specimens with a side length of 10 mm and height of 5 mm were fabricated on the Ti6Al4V substrate by selective laser melting (SLM). The gas-atomized TC4 powder with spherical particles and a size distribution ranging from 10 to 50 μm was used in this paper, as shown in Figure 1. The composition of the powder is shown in Table 1.

All parts were made with Renishaw AM 400 system equipment (Renishaw Plc., Gloucestershire, Britain), which has a 400 W ytterbium fiber laser (Renishaw Plc., Gloucestershire, Britain) with a focused beam diameter of 70 μm. Samples were produced with a scanning speed (v) of 1200 mm/s, a laser power (P) of 400 W, 60 μm hatch spacing (h) (the distance between two adjacent scan vectors), and a 30 μm layer thickness (t). Layers were scanned using a pulsed laser mode according to a zigzag pattern, which was rotated 90° between each layer. This parameter set was determined to obtain fully dense, high-quality Ti6Al4V products.

Heat treatments were executed in a vertical tube furnace, in an argon-protected atmosphere and with a heating rate of approximately 10 °C /min. The parameters of the process were carried out as shown in Table 2. In the table, AC means air cooling and WQ means water quenching. The heat treatment system refers to the current typical wrought titanium alloy heat treatment process [22,26,27].

Examination of the microstructure occurred after grinding with SiC grinding paper up to a fine 3000-grit size, and polishing with SiO_2_ suspension. To reveal the microstructure, samples were etched with a solution containing 50 mL of H_2_O, 25 mL of HNO_3_ and 5 mL of HF. Because of the anisotropic build process, two cross sections are considered. One is a side view parallel with the build direction, and the other is a top view, perpendicular to the build direction. Micrographs were taken using a conic xip-6A microscope. A Hitachi SEM SU5000 (Hitachi Ltd., Tokyo, Japan) was used for the examination of higher-resolution micrographs. The microhardness of TC4 alloy samples was tested using a DHV-1000Z-type micro-Vickers hardness tester (Shuangxu Ltd., Shanghai, China) with a load of 4.904 N (500 g) and a loading of 15 s (GB/T4340.1–1991). Phase identification of the SLM-formed TC4 alloy specimens before and after heat treatment was conducted with an X-ray diffractometer (XRD; Cu Ka) (Rigaku, Tokyo, Japan) from 30° to 85°, operated at 40 kV and 100 mA, with a step size of 0.02 and a dwelling time of 0.3 s per step.

## 3. Results and Discussion

### 3.1. Microstructure

**Figure 2:**
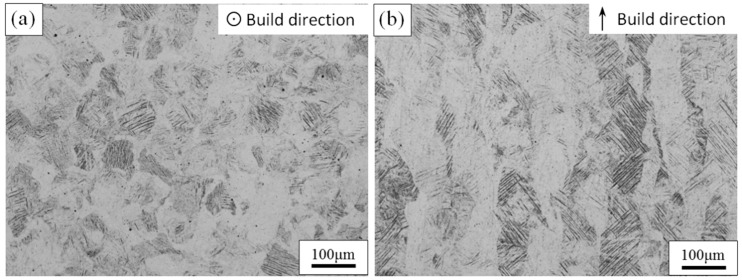
OM image of the top view (**a**) and the side (**b**) view of untreated TC4 produced by SLM.

Figure 2a,b show, respectively, a top and side view of non-heat-treated Ti6Al4V, produced by SLM. The microstructures of the top view and side view show completely different morphologies. The top view shows that equiaxed grains with an average diameter of 120 μm were developed in the SLM process. However, the macroscopic structures of the side view consist of columnar grains that grow epitaxially through multiple cladding layers with an average width of 120 μm, as shown in Figure 2b. According to the theory of constitutional supercooling by Chalmers [30], in order to use the alloy orientation to obtain planar crystals, following criteria that is supported to be satisfied,

(1) $$\frac{G_{L}}{R}\geq \frac{m_{0}c_{0}(k_{0}-1)}{k_{0}D_{L}}=\frac{\Delta T_{0}}{D_{L}}.$$

In this equation, *G_L_* is the liquid phase temperature gradient (K/mm) at the front of the solidification interface; *R* is the interface growth rate (mm/s); *m*_0_ is the liquidus slope; *C*_0_ is the alloy average composition; *K*_0_ is the equilibrium solute distribution coefficient; *D_L_* is the solute diffusion coefficient; Δ*T*_0_ is the equilibrium crystallization temperature interval. The solid–liquid interface morphology in directional solidification is determined by *G_L_*/*R*. When the liquid phase temperature gradient *G_L_* of the front of the solidification interface is larger, the solid–liquid interface tends to be smoother. In this situation, the crystal grains advance in the opposite direction to the heat flow. Finally, the columnar crystals grow in a certain direction [31].

The main axis of the columnar crystals is perpendicular to the deposition direction, and overlapping tracks in the as-built sample are not obvious. In the side view, the macroscopic structure presents the alternating light and dark growth, which is mainly due to the different crystal orientations. The main axis of the columnar crystal through the cladding layers is perpendicular to the scanning direction of the laser beam. This is mainly due to the temperature gradient of the molten pool being essentially perpendicular to the scanning direction during the SLM process, and the bottom of the molten pool is solidified first. The tops of the columnar crystals that have already solidified are re-melted when the laser beam scans the powder layers and the bottom of the unfused columnar crystal becomes the nucleus of the directionally solidified layer. Consequently, the epitaxial growth of the columnar crystal continues in accordance with the deposition direction [32].

**Figure 3:**
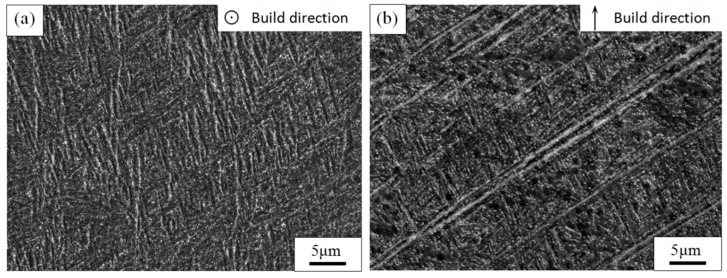
SEM images show the microstructure of untreated TC4 produced by SLM. (**a**) The microstructure of top view; (**b**) the microstructure of side view.

**Figure 4:**
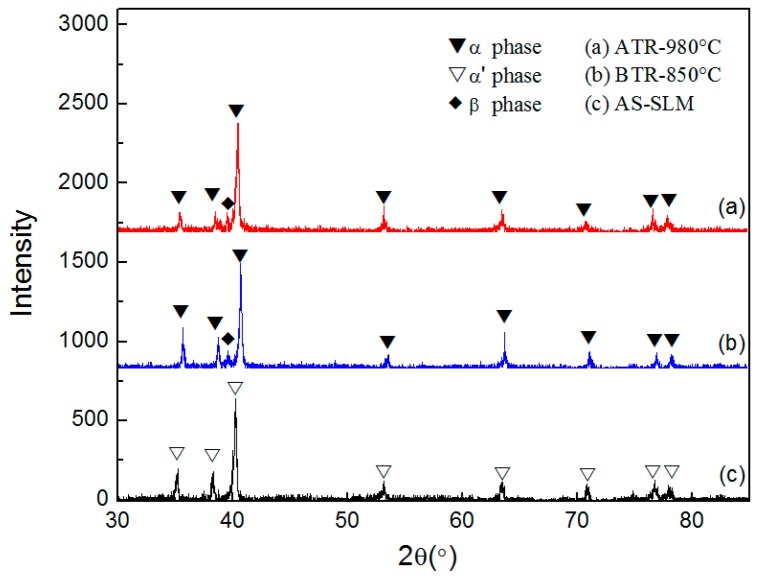
XRD analysis results of SLM formed TC4 before and after heat treatment.

The as-built sample’s SEM microstructures of TC4 alloy manufactured by SLM are shown in Figure 3. Figure 3a displays the internal microstructure of the equiaxed grain in the top view observed by SEM. The interior of the equiaxed grains mainly consists of the acicular martensitic α′-phase. The columnar crystals in the side view largely comprise an extremely fine acicular martensite α′-phase with a certain orientation. The β-phase was not detected by XRD in the TC4 titanium alloy fabricated by SLM (Figure 4); moreover, Bey et al. also identified the absence of β-phase in as-built TC4 alloys [22].

The reason as to why the acicular martensitic α′-phase formed in the TC4 alloy manufactured by SLM is that the transient temperature of the TC4 alloy is extremely high under the action of a laser. This preferentially forms the β-phase, and then forms a non-diffusion shear α′-phase during subsequent rapid cooling. The cooling rate during SLM processing is greater than 410 Ks^−1^, which has been suggested to be the correct rate for martensite α′ formation [33]. The α′-phase is hexagonally close packed (HCP) and maintains a Burgers relationship with the β-phase of the body-centered cubic (BCC): (110) β//(001) α; (111) β//(1120) [22].

### 3.2. Influence of Temperature

#### 3.2.1. Sample Annealed in the BTR

**Figure 5:**
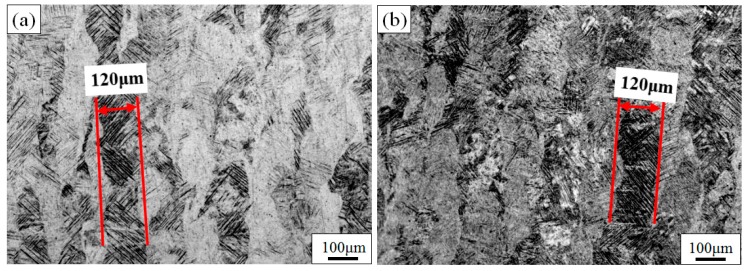
OM images show the etched morphology of TC4 SLM part after heat treating at different temperatures for 4 h, followed by AC. (**a**) 550 °C and (**b**) 750 °C below the *T*_0_.

**Figure 6:**
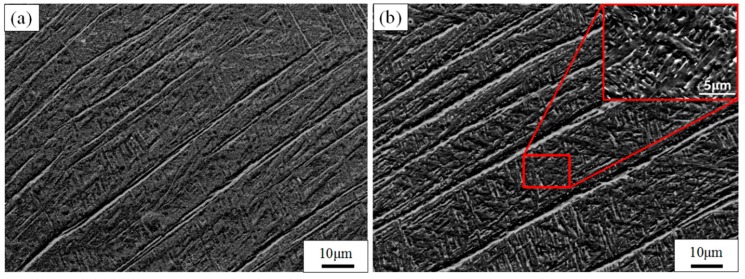
SEM images show the microstructure of TC4 SLM part after heat treating at different temperatures for 4 h, followed by AC. (**a**) 550 °C and (**b**) 750 °C below the *T*_0_.

The morphology of the SLM material after heat treatment at different temperatures in the BTR is shown in Figure 5. The dark regions indicate the etched β-phase. The long, columnar grains remain visible in the side view of the material at different annealing temperatures, and the width of grain is still approximately 120 μm. Macroscopically, the sample after 550 °C heat treatment has almost no change; however, the sample treated in 750 °C is darker than the OM photograph before heat treatment. This is because a lamellar α + β mixture has been formed after treatment at 750 °C but not 550 °C, as can be clearly seen in Figure 6. This observation corresponds to the α-dissolution temperature *T*_diss_, defined by Kelly as 705 °C [34], at which the α-phase starts to dissolute into the β-phase under equilibrium heating conditions. After annealing in the BTR, lower hardness but better plasticity are obtained, due to the lamellar α + β mixture [35].

Microstructures of the TC4 SLM part after heat treatment at different temperatures are shown in Figure 6. As shown in Figure 6a,b, when the residence time is 4 h and the annealing temperature is increased from 550 to 750 °C, the width of the acicular α-phase increases from 0.8 to 2.3 μm. After 4 h at 550 °C, the microstructure does not change significantly compared to the TC4 SLM part. This is because the rapid cooling of the as-built sample during the SLM forming process results in twinning dislocation inside of the alloy, which could be a reason for the poor ductility of the as-received alloy during tension [27]. These defects have somewhat of a hindering effect on the decomposition of the α′-phase. In addition, at 550 °C annealing, the phase decomposition driving-force is insufficient due to the low temperature. In BTR heat treatments, the growth of β-phase at α′ grain boundaries, as well as at internal twin boundaries started at *T*_diss_ and below *T*_0_ [35]. After 4 h at 750 °C, in contrast, the fine martensitic structure has been transformed into a mixture of α + β, in which the α-phase is present as fine needles (Figure 6b). This is because of the greater phase decomposition driving force at high temperatures, which promotes the decomposition of α′-phase. The width of α-phase in the alloy increases to 2.3 μm, as a result of aggregation and growth during the retaining at 750 °C.

#### 3.2.2. Sample Annealed in the ATR

**Figure 7:**
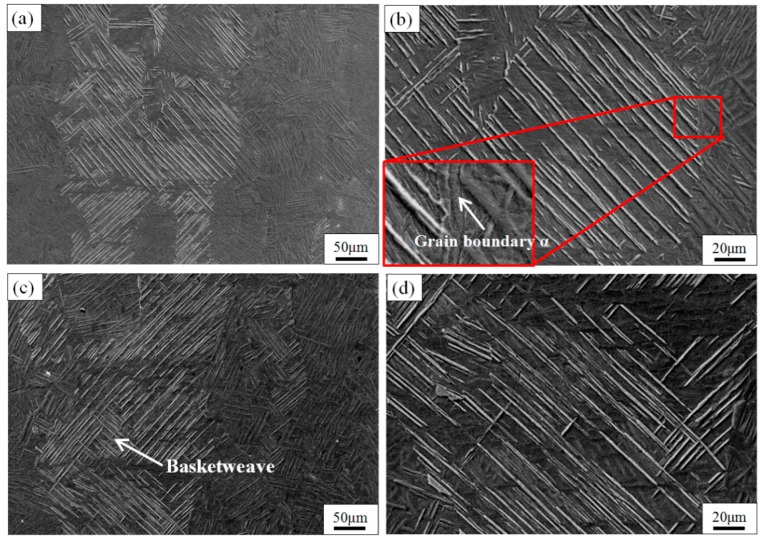
SEM images show the microstructure of TC4 SLM part after heat treatment at different temperatures of recrystallization annealing for 1 h, followed by WQ. The side view of (**a**) 900 °C and (**c**) 980 °C above the *T*_0_; (**b**,**d**) show an enlarged view of the columnar grains of (**a**,**c**), respectively.

Microstructures of the TC4 SLM part after heat treatment at different temperatures in the ATR are shown in Figure 7, in which the light plates indicate primary α-phase. Figure 7a,c show the side view of the SLM material after heat treatment at 900 and 980 °C, respectively. It can be seen that the length of the prior β grains seems unchanged but the width has increased and is now roughly 250 μm wide after 1 h at both 900 and 980 °C. This is because the higher temperature provides more driving force for the α-phase to decompose into the β-phase during the high temperature holding, and the adjacent β grow and connect with each other. The XRD analysis results still show an α + β microstructure, whereas a large amount of the transformed β structure was expected. This suggests that the β-transus of the SLM material is higher than 980 °C.

**Figure 8:**
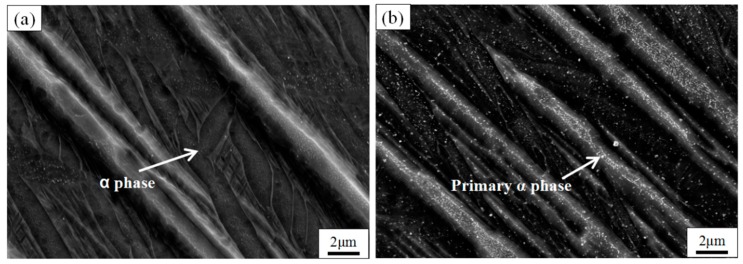
SEM images show the microstructure of TC4 SLM part after heat treating at different temperatures of recrystallization annealing. (**a**) 900 °C/1 h/WQ; (**b**) 980 °C/1 h/WQ.

As shown in Figure 7b,d, when the temperature is increased from 900 to 980 °C, the plates primary α-phase width inside each of the columnar grains is both about 2.5 μm, because the existence of the β-phase hinders the growth of the primary α-phase [20], which is derived from the martensite α′-phase and surrounded by β-phase. The length of the primary α-phase at 900 °C can reach around 300 μm and at 980 °C it is mostly 200 μm. However, after 1 h at 980 °C (Figure 7d), the number of primary α-phases increases, and some small segments of the primary α-phases with a length of 20–100 μm are also formed between the α-phases. The cause of the above phenomenon is that the driving-force of α′-phase decomposition is stronger during the holding process at 980 °C. During the holding process, the higher the temperature, the smaller the volume fraction of α-phase and the larger the volume fraction of β-phase [26]; then, during the water quenching process, the β-phase precipitates and transformed to α′-phases on the dislocations and along the substructural boundaries of the primary α [36], so that they are separated into small segments. Thus, the long plates shown in Figure 8 are composed of primary α-phase, and spaces between them are filled by α- and α′-phases. This can be seen clearly in Figure 8. When the segments develop with a certain orientation, a fine basketweave structure finally emerges [37]. The mechanical properties of the basket comprise high-temperature properties such as creep behavior deformation and stress rupture properties, better fracture properties and resistance to crack growth, but poor plasticity and thermal stability [19]; therefore, microhardness is significantly increased. As shown in the enlarged view of the red box in the lower left corner of Figure 7b, it can be seen that the α-phase grain boundary is formed outside of the columnar grains after the 900 °C treatment. It is the hindrance of the α-grain boundaries that causes the columnar grains to be connected with each other without limitation. Figure 8 is an enlarged image of the SEM, showing the microstructure of TC4 produced by SLM at different annealing temperatures in the ATR. It can be seen that the secondary α-phase and the coarse primary α-phase are formed after the TC4 SLM part is treated at both 900 and 980 °C. Through a comparison of Figure 8a,b, it is clear that an increased β-phase, and a basketweave microstructure is formed in the β columnar grains of the sample after heat treatment at 980 °C; this is advantageous for increasing the sample’s microhardness, but also lowers its plasticity [27].

### 3.3. Influence of the Residence Time

**Figure 9:**
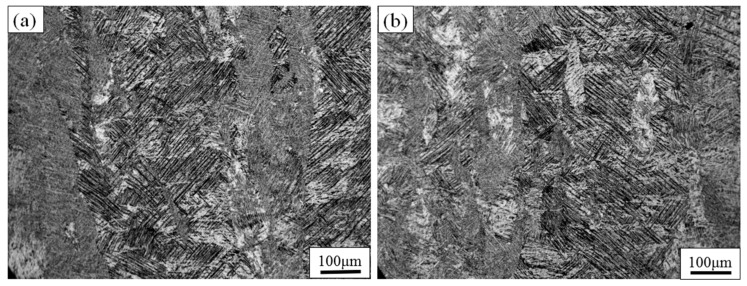
OM images show the etched morphology of TC4 SLM part after heat treating at (**a**) 2 h and (**b**) 4 h under 850 °C, followed by air cooling.

Figure 9a,b shows, respectively, the OM image of TC4 produced by SLM at 850 °C for 2 h and 850 °C for 4 h, followed by air cooling. When the temperature rises to 850 °C, the β-phase fraction is increased compared with the OM image at 750 °C [26]. With the increase of the residence time from 2 h to 4 h, the β-phase fraction is almost unchanged. This is because the heat treatment temperature is between *T*_diss_ and *T*_0_, and the coexistence of the α- and β-phases causes them to hinder each other. If the heat treatment temperature is above the β-transus, growth of the β-phase will be without hindrance and rapid [17].

**Figure 10:**
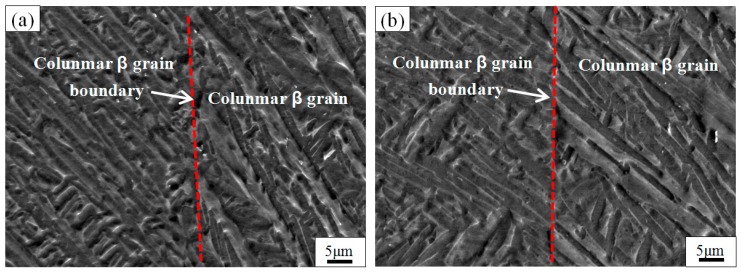
SEM images show the microstructure of TC4 SLM part after heat treatment at different residence time. (**a**) 850 °C/2 h/AC; (**b**) 850 °C/4 h/AC.

Figure 10a,b show respectively the SEM microstructure of TC4 produced by SLM at 850 °C for 2h and at 850 °C for 4 h, followed by air cooling. The microstructures of the alloys comprise a coarse mixture (α + β), and the widths of the plate α-phases obtained after 2 h and 4 h of holding at these temperatures are approximately 2.8 and 3.1 μm, respectively. Therefore, the effect of residence time on the growth of the α-phase is not significant, but it is significant compared to the α-phase of TC4 produced by SLM at 750 °C for 4 h, followed by air cooling. The dispersive distribution of β-phase obtained through decomposition of the α′-phase and the α-phase and the β-phase hinder each other; thus, the effect of residence time on the size of grains and precipitated phases is small. Huang et al. similarly found that the α′-phase of TC4 produced by SLM was completely decomposed to the α-phase when held at 800 °C for 2 h and the α-phase will grow by a certain degree when the holding is continued [38]. The microhardness of the sample obtained from different residence times is almost the same due to no significant changes in the microstructure during different heat treatment holding times.

### 3.4. Microhardness

**Figure 11:**
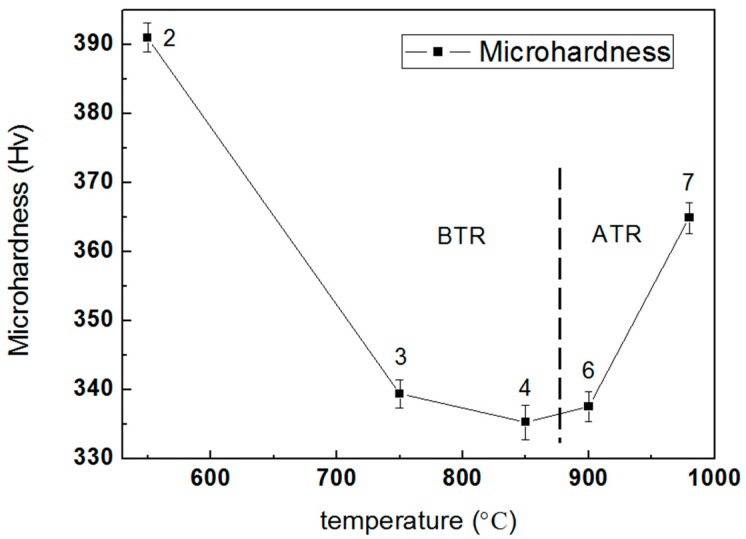
The microhardness curves of TC4 SLM part after heat treating at different temperatures. The number in the curve is equivalent to the sample number in Table 2.

Figure 11 shows the microhardness curves of the TC4 alloy produced by SLM at different heat treatment temperatures. In terms of the annealing temperature in the BTR (left of the dashed line), the microhardness of the TC4 alloy gradually decreases with increasing temperature, but the slope becomes less steep. For the annealing temperature in the ATR (right side of the dashed line), as the heat treatment temperature increases, there is a significant increase in microhardness. The higher cooling rate during the water quenched process after treatment at the ATR temperature causes the hardness to be improved. The microhardness of the as-built sample is 381.3 Hv, which is lower than Point 2, as did work by Wu et al. [27]. In addition, the hardness of samples treated at 850 °C for 2 h with air cooling and for 4 h at the same condition is almost equal, indicating that the effect of holding time on hardness is not significant.

It is evident that the change of hardness with post-heat-treatment temperature is strictly related to the microstructural change of the as-built TC4 specimens. At the BTR temperature, as the temperature exceed T_diss_, the relatively soft β-phase fraction increases as the temperature increases; this is because the original martensites decompose more thoroughly, thus decreasing the hardness. Because of the insufficient phase decomposition driving-force, α-martensite on twin boundaries and dislocations inside its plates transformed into α-phases partially and formed a refinement substructure, resulting in a high microhardness at Point 2. At Point 4, complete decomposition of α′-martensite to an α + β mixture caused the turning point of the microhardness curve. In the ATR heat treatment stage, a martensitic transition will recur in the high-temperature stable β-interlayers during water quenching, and in the obtained basketweave microstructure that also plays a role in strengthening hardness.

## 4. Conclusions

1. The side view of non-heat-treated TC4, produced by SLM, reveals long columnar grains which grow through multiple cladding layers and are oriented in the building direction. The extremely high cooling rate during the SLM process leads to the formation of a fine acicular martensite α′-phase with a certain orientation inside of the columnar grains.
2. Following heat treatment in the BTR, the internal acicular martensite α′-phase of the SLM TC4 part is converted into the α-phase and forms a lamellar α + β mixture, which gradually increases in structure size with increasing temperature. After being annealed in the ATR, the β-phase that formed in the holding process is transformed to martensite α during the cooling process and a fine basketweave structure emerges, which improves the microhardness of the alloy. At the BTR annealing temperature, the holding time has little effect on the microstructure and properties.
3. At the BTR annealing temperature, the microhardness of SLM-formed TC4 alloy gradually decreases with increasing temperature. However, the microhardness increases significantly with increasing temperature when the TC4 SLM part is treated under ATR annealing.

## Figures and Tables

**Table 1:** Composition of the TC4 alloy powder (wt %).

C	O	N	H	Al	V	Fe	Ti
≤0.03	≤0.1	≤0.01	≤0.002	6.0–6.75	3.5–4.5	≤0.20	Bal.

**Table 2:** Heat treatments of the TC4 sample produced by SLM.

Sample	Temperature Region	Temperature (°C)	Residence Time (h)	Cooling Rate *
1	BTR	As-built	-	-
2		550 °C	4 h	AC
3		750 °C	4 h	AC
4		850 °C	4 h	AC
5		850 °C	2 h	AC
6	ATR	900 °C	1 h	WQ
7		980 °C	1 h	WQ

* AC = Air cooling; WQ = Water quenching.
